# Molecular imaging of oxidative stress using an LED-based photoacoustic imaging system

**DOI:** 10.1038/s41598-019-47599-2

**Published:** 2019-08-06

**Authors:** Ali Hariri, Eric Zhao, Ananthakrishna Soundaram Jeevarathinam, Jeanne Lemaster, Jianjian Zhang, Jesse V. Jokerst

**Affiliations:** 1 Department of NanoEngineering, University of California, San Diego, 9500 Gilman Drive, La Jolla, CA 92093 USA; 2 Materials Science and Engineering Program, University of California, San Diego, 9500 Gilman Drive, La Jolla, CA 92093 USA; 3 Department of Radiology, University of California, San Diego, 9500 Gilman Drive, La Jolla, CA 92093 USA; 4 Key Laboratory of Synthetic and Natural Functional Molecule Chemistry of the Ministry of Education, Modern Separation Science Key Laboratory of Shaanxi Province, College of Chemistry & Materials Science, Northwest University, Xi’an, China

**Keywords:** Molecular imaging, Ultrasound

## Abstract

LED-based photoacoustic imaging has practical value in that it is affordable and rugged; however, this technology has largely been confined to anatomic imaging with limited applications into functional or molecular imaging. Here, we report molecular imaging reactive oxygen and nitrogen species (RONS) with a near-infrared (NIR) absorbing small molecule (CyBA) and LED-based photoacoustic imaging equipment. CyBA produces increasing photoacoustic signal in response to peroxynitrite (ONOO^−^) and hydrogen peroxide (H_2_O_2_) with photoacoustic signal increases of 3.54 and 4.23-fold at 50 µM of RONS at 700 nm, respectively. CyBA is insensitive to OCl^−^, ˙NO, NO_2_^−^, NO_3_^−^, tBuOOH, O_2_^−^, C_4_H_9_O˙, HNO, and ˙OH, but can detect ONOO^−^ in whole blood and plasma. CyBA was then used to detect endogenous RONS in macrophage RAW 246.7 cells as well as a rodent model; these results were confirmed with fluorescence microscopy. Importantly, CyB suffers photobleaching under a Nd:YAG laser but the signal decrease is <2% with the low-power LED-based photoacoustic system and the same radiant exposure time. To the best of our knowledge, this is the first report to describe molecular imaging with an LED-based photoacoustic scanner. This study not only reveals the sensitive photoacoustic detection of RONS but also highlights the utility of LED-based photoacoustic imaging.

## Introduction

Reactive oxygen and nitrogen species (RONS) modulate important functions in living systems. Endogenous RONS facilitate signal transduction^1^, smooth muscle relaxation, and blood pressure modulation^2^. Dysregulated RONS can lead to diseases such as cancer^3–5^, and RONS detection is useful in the diagnosis and treatment of infections and disease. There are various fluorescent contrast agent to detect and quantify RONS^2,6,7^, but their *in vivo* applications are limited due to the low spatial resolution and limited penetration depth of fluorescence^8^.

Photoacoustic imaging (PAI) is an alternative imaging approach and combines optical and ultrasound imaging features^9–11^ and offers better penetration with less scatter than fluorescent imaging. Smart activatable photoacoustic probes can produce photoacoustic signal in the presence of specific molecules or events such as RONS^12,13^. Pu and workers have described several RONS-sensitive molecules with activatable photoacoustic signal^8,14–17^ including Cheng *et al*. who reported CyBA for detection of RONS using fluorescence imaging^18^.

Regardless of the imaging target, PAI usually uses high energy lasers. Although these lasers offer tunable excitation wavelengths and high power, they are also large and cumbersome and require regular maintenance. In addition, many contrast media are not stable at such high fluences. In one example, the photoacoustic signal of gold nanorods decreased by ~30% after exposure to 120 µs of 9 mJ/cm^2^ fluence)^8^ due to a change in the nanorod morphology^19^. In another example, Onoe *et al*. developed a symmetrical NIR cyanine dye for cancer imaging utilizing fluorescence and photoacoustic imaging^20^, but this probe degraded under high-energy laser pulses during imaging^21^.

Although a more stable probe design is an obvious solution, one exciting alternative is pulse laser diodes (PLDs)^22^ or light emitting diodes (LEDs)^23,24^. The LED fluence is on the order of μJ and 1000-fold lower than high energy lasers^25^. We previously described a commercially available LED-based photoacoustic imaging system and validated it with phantoms and cellular imaging^25^. Here, we report the use of a near-infrared (NIR) small molecular probe for photoacoustic imaging that is sensitive to various types of RONS^18^. We first evaluated the probe with both a laser and a LED. The absorption and photoacoustic response of this probe were examined with different RONS in different sample matrices such as PBS/DMSO, plasma, and fresh blood. We then used the probe to measure the endogenous RONS produced stimulated macrophages with the LED-based photoacoustic imaging system along with *in vivo* imaging of an inflammation model.

## Methods and Materials

### Chemicals

Aqueous PBS stock solution and hydrogen peroxide (H_2_O_2_) (30 wt%, Cat. #H325) were purchased from Thermo Scientific (Waltham, MA, USA). We purged PBS with N_2_ for 1 hour before any dilution and measurement. Peroxynitrite (ONOO^−^) was purchased from EMD Millipore Co. (Cat. #516620, MA, USA). Superoxide (O_2_^−^), Hypochlorite (OCl^−^), Nitric oxide (˙NO), Tert-butyl hydrogen peroxide (tBuOOH), Tert-butoxy radical (C_4_H_9_O˙), Nitrite (NO_2_^−^), Nitrate (NO_3_^−^), and Nitroxyl (HNO) were prepared by direct dilution of potassium superoxide (Cat. #278904), sodium hypochlorite (Cat. #239305),Tert-butyl hydroperoxide (Cat. #416665), Tert-butyl peroxide (Cat. #168521), Sodium nitrite (Cat. #237213), Sodium nitrate (Cat. #221341), and angeli’s salt (Cat. #176695). These were purchased from Sigma-Aldrich (Atlanta, GA, USA). Hydroxyl radicals (˙OH) were generated via the Fenton reaction between H_2_O_2_ and iron (II) perchlorate hydrate (Fe(ClO_4_)_2_) (Cat. #334081, Sigma-Aldrich, Atlanta, GA, USA). The 2′,7′-dichlorofluorescin diacetate (DCF-DA) (Cat. #D6883), N-acetylcysteine (NAC), Lipopolysaccharides (LPS) from *Escherichia coli*, and Zymosan A from Saccharomyces Cerevisiae were purchased from Sigma-Aldrich (Atlanta, GA, USA). A stock solution of the new synthesized dye was prepared by dissolving in dimethyl sulphoxide (DMSO) (Thermo Scientific, Waltham, MA, USA). Normal pooled human plasma was purchased from Innovative Research Inc. (Novi, MI, USA). Blood was obtained from healthy donors in 3.2% sodium citrate vacutainers (BD life Science) and informed consent was obtained from all subjects. All methods were performed in accordance with the relevant guidelines and regulations. All experimental protocols were approved by University of California San Diego.

### Probe synthesis and characterization

Cheng *et al*. first developed and reported the application of CyBA for detection of RONS using fluorescence imaging^18^. The synthesis was adopted from their study^18^ and used a mixture of compound CyOH (38 mg, 0.1 mmol) and Cs_2_CO_3_ (130 mg, 0.40 mmol) in anhydrous CH_2_Cl_2_ (10 mL) in a round-bottom flask was stirred for 20 min at room temperature. The 4-bromomethylphenylboronic acid pinacol ester (60 mg, 0.20 mmol) was then added and the reaction was further stirred overnight at room temperature under N_2_ atmosphere. The reaction mixture was then washed with water (30 mL) and extracted with CH_2_Cl_2_ (3 × 30 mL). The organic layer was dried with anhydrous Na_2_SO_4_ and concentrated under reduced vacuum. The pure product CyBA was obtained as a blue solid (45 mg, 75%) after purification by flash column chromatography (CH_2_Cl_2_/MeOH = 10:1). ^1^H NMR (300 MHz, CD_3_OD): *δ* = 8.70 (d, *J* = 15.0 Hz, 1H), 7.80 (d, *J* = 8.1 Hz, 2H), 7.68 (d, *J* = 7.2 Hz, 1H), 7.55 (m, 2H), 7.50 (d, *J* = 8.1 Hz, 2H), 7.45 (m, 2H), 7.34 (s, 1H), 7.02 (m, 2H), 6.49 (d, *J* = 15.0 Hz, 1H), 5.29 (s, 2H), 3.85 (s, 3H), 2.76 (t, *J* = 6.0 Hz, 2H), 2.71 (t, *J* = 6.0 Hz, 2H), 1.92 (m, 2H), 1.81 (s, 6H), 1.34 (s, 12H). MS of compound CyBA: calculated for C_39_H_43_BNO_4,_ [M^+^]: 600.39; observed MS: m/z 600.40.

### Instrumentation

We used a SpectraMax M5 spectrophotometer for absorbance measurements. An EVOS fluorescence microscope with FITC filter sets (Life Technologies Inc., Ca, USA) was utilized for brightfield and fluorescence microscopy imaging. Photoacoustic images were acquired using an LED-based photoacoustic imaging system from CYBERDYNE Inc. (formerly Prexion)^25^. The system is equipped with a 128-element linear array ultrasound transducer with a central frequency of 10 MHz and a bandwidth of 80.9% fitted with two 690 nm LED arrays. The repetition rate of these LEDs is tunable between 1, 2, 3, and 4 K Hz. The pulse width can be changed from 50 ns to 150 ns with a 5-ns step size. The transducer can be scanned to generate three-dimensional (3D) data using maximum intensity projection (MIP) algorithm.

### Tissue culture

RAW 246.7 cells (ATCC TIB-71) were cultured using Dulbecco’s Modified Eagle’s Medium (DMEM, Gibco) supplemented with 10% fetal bovine serum (Sigma) and 1% antibiotic-antimycotic (Thermo Fisher Scientific). Cells were incubated at 37 °C and media was replaced every 2–3 days. Cells were cultured until 90% confluence and passaged mechanically through scraping.

### Probe stability

A Nd:YAG laser with an optical parametric oscillator (Vevo 2100 photoacoustic scanner, Visualsonics) and a LED (LED-based scanner, CYBERDYNE Inc.) at 690 nm were used to evaluate the stability of the new probe. Here, 15 µL of a 0.1 mM solution were placed in polyethylene tubing (OD: 1.27 mm, ID: 0.85 mm) inside the chicken breast to scatter the incident light (at depth of 1 cm) and imaged with the laser or LED to study the stability of the probe. Laser illumination used a pulse width and frame rate of 5 ns and 6 Hz, respectively. A laser pyroelectric energy sensor (PE50BF-C, Ophir LLC, USA) measured the laser fluence. LED illumination used a LED-based scanner (pulse width: 70 ns, frame rate: 6 Hz) with a photodiode sensor (S120C, Thorlabs Inc., USA) to measure the LED fluence. The effective illumination for both is 16 μs (effective illumination = number of laser or LED pulses × the pulse width).

### Absorption response of CyBA toward RONS

We first studied the absorption response of the probe toward RONS. We used various concentrations for ONOO^−^ (12.5, 25, 50, 75, 100, and 200 µM) to evaluate changes in the probe’s absorption spectrum (400 nm – 800 nm) in the presence of RONS. Next, we tested the effect of OCl^−^, ˙NO, NO_2_^−^, NO_3_^−^, tBuOOH, O_2_^−^, C_4_H_9_O˙, HNO, ˙OH, H_2_O_2,_ and ONOO^−^ by monitoring the change in 700 nm absorption using a 80 µM solution of the probe and 50 µM RONS. Three replicates were used.

### Photoacoustic response of CyBA in the presence of RONS

CyBA and CyOH (activated form of the probe) were studied at 20, 40, 80, 160, 320, and 640 µM. We also examined the photoacoustic response of CyBA to 50 µM of OCl^−^, ˙NO, NO_2_^−^, NO_3_^−^, tBuOOH, O_2_^−^, C_4_H_9_O˙, HNO, ˙OH, H_2_O_2_, and ONOO^−^. Various concentrations of ONOO^−^ and H_2_O_2_ to measure the sensitivity of the probe: 12.5, 25, 37, 50, 75, 100, and 200 µM ONOO^−^; 31, 62, 125, 250, 2500, 5000 µM, and 50 mM H_2_O_2_. All samples were placed inside Teflon light wall tubes and scanned with the transducer (the scan size is 10 mm) to generate three-dimensional (3D) data using maximum intensity projection (MIP) algorithm.

### Photoacoustic response of the probe towards ONOO^−^ in pooled human plasma and blood

We extended the work with ONOO^−^ to normal pooled human plasma and whole human blood. First, we evaluated the absorption response of 125 µM probe in the presence of 25, 50, 125, 185, 250, 375, and 500 µM of ONOO^−^ in normal human plasma. Next, we used LED-based photoacoustic system to study the response of the probe towards ONOO^−^ at 25, 37.5, 50, 75, and 100 µM in normal pooled human plasma and blood. Finally, we calculated a detection limit of ONOO^−^ in plasma and blood.

### *In vitro* fluorescence imaging of RONS

RAW 246.7 cells were plated into 6-well plates (50,000 cells/well) and incubated overnight. Cells were washed once with PBS, and 2 mL of media was added; 20 μL of LBS in PBS was added to the respective groups (final concentration: 1 μg/mL), and the plates were incubated for 4 hours. Then, 20 μL of NAC in PBS was added to the respective groups (final concentration: 0.1, 1, or 10 mM) and incubated for 1 hour. Finally, 20 μL of DCF-DA in DMSO was added to all groups (final concentration: 20 μM) and incubated for 20 minutes. Cells were imaged with a fluorescence microscope (EVOS) using a GFP filter.

### *In vitro* photoacoustic imaging of RONS

RAW 246.7 cells were plated into 6-well plates (1 million cells/well) and incubated overnight. Cells were washed once with PBS and 1 mL of media was added. 10 μL of LPS was added to the respective groups (final conc. 10 μg/mL) and incubated for 4 hours. Then, 10 μL of NAC was added to the respective groups (final concentration: 0.1 or 10 mM) and incubated for 1 hour. Next, 400 μL of CyBA was added to the respective groups (final concentration: 125 μM) and incubated for 30 minutes. Cells were mechanically detached, centrifuged at 1000 g for 5 minutes, and the supernatant was removed. All cells were resuspended in 50 μL PBS and imaged at 690 nm using LED scanner. As an additional control, LPS and CyBA were mixed without any cells and imaged.

### *In vivo* photoacoustic imaging evaluation of CyBA

All animal experiments were performed in accordance with NIH guidelines and approved by the Institutional Animal Care and Use Committee (IACUC) at the University of California, San Diego. We utilized CyBA for the *in vivo* photoacoustic imaging of RONS. Zymosan was applied to induce the acute edema in a murine model. Zymosan (20 mg/ml) was injected intramuscularly into the right biceps femoris muscle of the upper hind limb (n = 3). After 20 minutes incubation, CyBA (160 μM in saline) was injected at same location. We monitored the photoacoustic intensity (690 nm as a wavelength) of the probe at the injection area in 0, 10, 20, 30, 45, 60, 90 minutes after injection. We also injected only Zymosan and only CyBA at the same location and monitored the photoacoustic intensity as a control.

### Statistical analysis

All photoacoustic data were analyzed with ImageJ (Bethesda, MD, USA)^26^. All raw images were converted to 8-bit images, and then the mean value and standard deviation of the photoacoustic intensity for ten regions of interest (ROI) per tube were calculated. The error bars represent the standard deviation; P values < 0.05 were considered to be significantly different.

## Results

**Figure 1:**
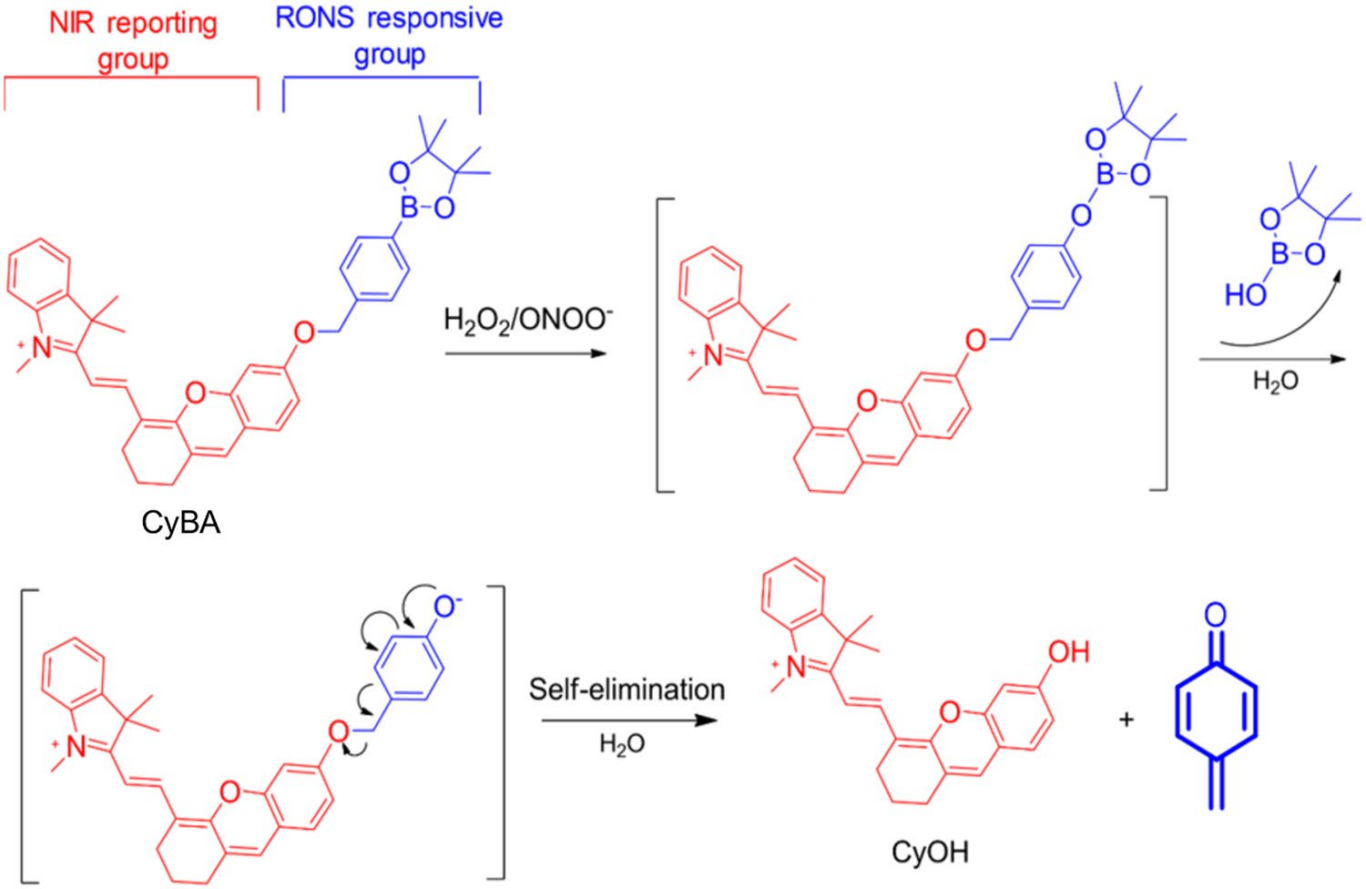
Design and mechanism of the probe (CyBA) for RONS imaging. The RONS species liberates the boronic acid group leading to increased absorbance of the cyanine core leading to increased photoacoustic signal. Adapted from Cheng *et al*.^18^.

CyBA is designed by conjugating a boronic acid-based self-immolative group to CyOH. CyBA is initially in a “caged” state with diminished electron-donating ability of the oxygen atom. Upon treatment with H_2_O_2_ or ONOO^−^, CyBA undergoes rapid oxidative cleavage of the borate ester moiety, followed by 1,6-elimination, eventually leading to free CyOH dye (“uncaged” state). The CyOH product has enhanced electron-donating ability from the oxygen atom, which makes its characteristic maximum absorption change in relative to CyBA. Thus, the probe enables photocoustic imaging of RONS (Fig. 1). We used mass spectrometry to show the decomposition of probe under high concentrations of RONS (peroxynitrite) and evaluation of CyBA/CyOH composition stability under our imaging conditions. Mass spectrometry data confirmed the stability of CyBA under LED illumination and confirmed that the reaction product of CyBA in presence of RONS (peroxynitrite) is indeed CyOH (Fig. S1).

### Probe stability

**Figure 2:**
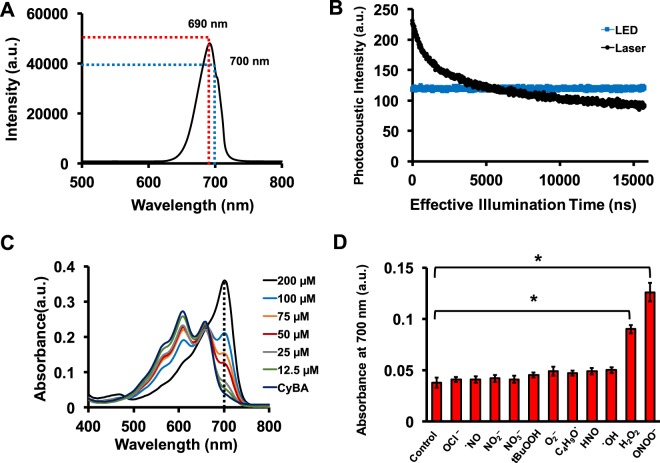
Absorption response of new probe toward RONS. (**A**) Spectral profile of the LED used for photoacoustic imaging. The spectrum has a peak at 690 nm and includes 700 nm. (**B**) Photoacoustic intensity of the probe under laser and LED illumination. The photoacoustic intensity decreases 2.6-fold under laser source but <2% with the LED. Therefore, the probe is stable when used with a LED-based photoacoustic imaging system. (**C**) The absorption spectra of the dye (80 µM) after addition of ONOO^−^ 12.5, 25, 50, 75, 100, and 200 µM. The dye has two absorption peaks around 660 and 610 nm. However, in the presence of RONS, the 700 nm peak appears and other peaks vanish. (**D**) Absorption of CyBA in the presence of 50 µM OCl^−^, ˙NO, NO_2_^−^, NO_3_, tBuOOH, O_2_^−^, C_4_H_9_O˙, HNO, ˙OH, H_2_O_2,_ and ONOO^−^_._ The control is PBS, and the error bars represent the standard deviation of three replicate measurements. ^*^Indicates p-value < 0.05.

The probe has a broad absorption peak at 700 nm that increases when activated by the RONS, but we used 690 nm excitation because that equipment was available in our lab. Figure 2A shows the spectral profile of the LED used in the photoacoustic imaging system. It has a peak at 690 nm (range: 625–725 nm); the intensity at 700 nm is ~65% of the maximum. The laser and LED intensity at 690 nm is 12.65 ± 0.65 mJ/cm^2^ and 5.5 μJ/cm^2^, respectively, i.e., the laser intensity is ~2000-fold higher than the LED.

Next, we studied the photoacoustic signal of the probe with Nd:YAG laser and LED in the absence of RONS. Figure 2B shows that the photoacoustic signal degraded 2.6-fold after 16 μs of effective time laser illumination (laser: 3150 pulses (at 20 Hz) × 5 ns pulse width = 16 μs; LED: 240 pulses (at 4000 Hz) × 70 ns = 16 μs). The LED system shows <2% signal loss. Thus, we used the LED-based photoacoustic imaging system to evaluate the probe in all following experiments.

### Absorption response of the probe toward RONS

Next, we studied the change in the probe absorption in the presence of RONS. Figure 2C shows the absorption spectra of the dye (80 µM) with and without ONOO^−^. The probe has two absorption peaks at 610 and 660 nm. The probe exhibited a new absorption peak at 700 nm upon addition of 12.5, 25, 50, 75, 100, and 200 μM ONOO^−^ (the peaks at 610 and 660 nm decreased). Only the 700 nm peak is seen at 200 μM ONOO^−^. Figure 2D shows the absorption response of the probe in the presence of OCl^−^, ˙NO, NO_2_^−^, NO_3_^−^, tBuOOH, O_2_^−^, C_4_H_9_O˙, HNO, ˙OH, H_2_O_2,_ and ONOO^−^ at 700 nm using 80 µM dye and 50 µM RONS. The absorption increased 2.38 and 3.31-fold in the presence of ONOO^−^ and H_2_O_2_, respectively (p-value < 0.05).

### Photoacoustic response of the probe in the presence of RONS

**Figure 3:**
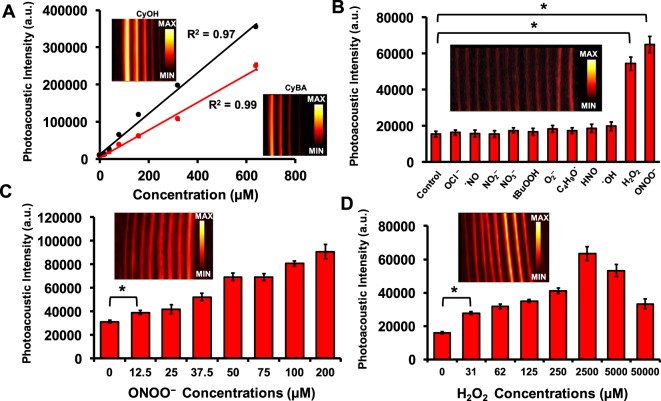
Photoacoustic response of the probe in presence of RONS. **(A**) Photoacoustic intensity as function of different probe concentrations. Linear correlation is observed between photoacoustic intensity and new probe concentration when imaged with the LED system. (**B**) Photoacoustic signal of 80 µM probe in the presence of 50 µM of OCl^−^, ˙NO, NO_2_^−^, NO_3_^−^, tBuOOH, O_2_^−^, C_4_H_9_O˙, HNO, ˙OH, H_2_O_2,_ and ONOO^−^. The inset shows the MIP photoacoustic image of all the samples. The control is PBS. Dose response curve of probe to ONOO^−^ (**C**) and H_2_O_2_ (**D**); insets are the MIP images. Error bars represent multiple ROIs per tube. *Indicates p-value < 0.05.

Figure 3A shows the photoacoustic intensity as function of CyBA and CyOH concentration. The insets show the photoacoustic MIP images corresponding to CyBA and CyOH. A linear correlation was observed between the photoacoustic intensity and both CyBA (R^2^ = 0.99) and CyOH (0.97) concentrations. We used 80 µM to examine the photoacoustic response to OCl^−^, ˙NO, NO_2_^−^, NO_3_^−^, tBuOOH, O_2_^−^, C_4_H_9_O˙, HNO, ˙OH, H_2_O_2,_ and ONOO^−^. This concentration was used because it has relatively low signal in the absence of RONS. Figure 3B shows that the probe is insensitive to OCl^−^, ˙NO, NO_2_^−^, NO_3_^−^, tBuOOH, O_2_^−^, C_4_H_9_O˙, HNO, and ˙OH, but the signal increased 3.54 and 4.23-fold with 50 µM H_2_O_2_ and ONOO^−^, respectively. Figure 3C,D detail the signal as a function of ONOO^−^ and H_2_O_2_ concentrations. Higher concentrations lead to more absorption and thus more photoacoustic signal with a detection limit of 12.5 µM ONOO^−^ (p-value < 0.05). Similarly, the H_2_O_2_ sensitivity is 31 µM.

### Utility of the probe in pooled human plasma and blood

**Figure 4:**
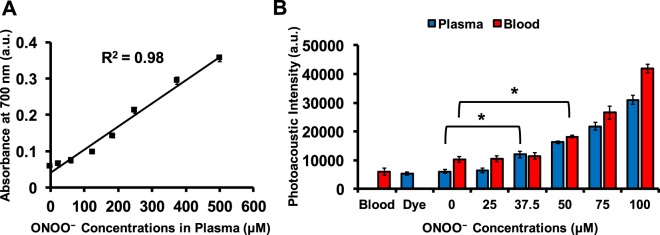
Photoacoustic signal as a function of ONOO^−^ in normal pooled human plasma and whole blood. (**A**) Linear absorbance response of 125 µM probe in pooled human plasma at 700 nm with various ONOO^−^ concentrations. (**B**) Photoacoustic response of 125 µM probe in human plasma and fresh human blood to ONOO^−^ from 0 to 100 µM. The ONOO^−^ detection limits are 37.5 and 50 µM in plasma and blood, respectively. *Indicates p-value < 0.05.

Figure 4A shows a linear correlation (R^2^ = 0.98) between absorbance at 700 nm and ONOO^−^ concentrations in pooled human plasma. Figure 4B shows the photoacoustic response of the probe in the presence of ONOO^−^ in pooled human plasma and fresh whole blood. The detection limits of ONOO^−^ in plasma and blood were 37.5 and 50 μM, respectively (p-value < 0.05). The detection limit is higher in blood because hemoglobin and deoxyhemoglobin increase the background signal.

### *In vitro* characterization of endogenous RONS

**Figure 5:**
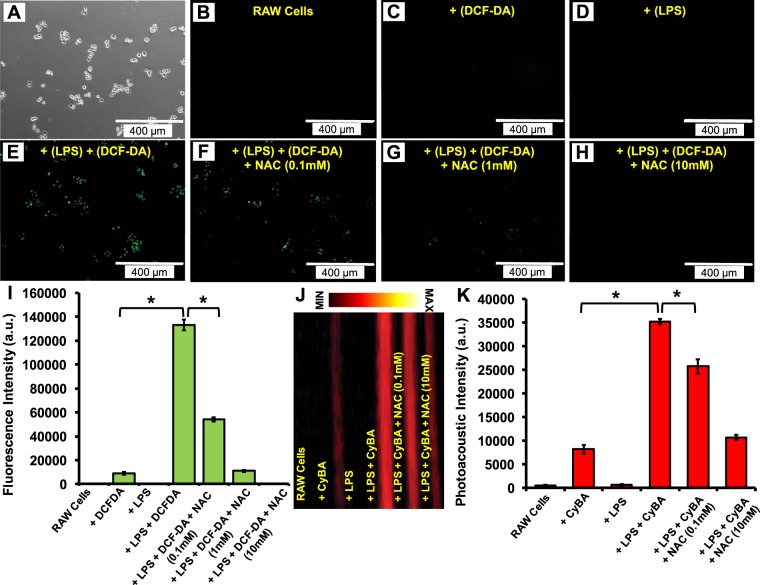
*In vitro* characterization of endogenous RONS. (**A**) Brightfield microscopy image of RAW 264.7 cells line. (**B**) Fluorescence microscopy image of RAW 264.7 cells. Fluorescence response of (**C**) DCF-DA (20 µM) after incubation with RAW cells, (**D**) LPS (1 μg/mL) after incubation with RAW cells, (**E**) LPS (1 μg/mL) and DCF-DA (20 µM) after incubation with RAW cells. Green signal indicated the presence of RONS in these cells after incubation with LPS. Fluorescence response of LPS and DCF-DA incubated with RAW cells after treating (**F**) 0.1 mM NAC, (**G**) 1 mM NAC, and (**H**) 10 mM NAC. (**I**) Quantitative analysis of fluorescence intensity in all samples in (**B**–**H**). The RONS indicator DCF-DA shows increased fluorescence with LPS stimulation. A NAC RONS scavenger decreases fluorescence. (**J**) The MIP photoacoustic image of RAW cells, RAW cells with the probe (+CyBA), RAW cells with LPS (+LPS), RAW cells with LPS and CyBA (+CyBA + LPS), and RAW cells incubated with LPS/CyBA and various concentrations of NAC (0.1 and 10 mM). (**K**) Quantitative analysis shows increased photoacoustic signal in the presence of probe and RAW cells incubated with LPS. This suggests RONS generation after monitoring using LED based photoacoustic imaging. *Indicates p-value < 0.05.

We next tested the ability of the probe to measure endogenous RONS produced by murine macrophage RAW 264.7 cells^27–29^. Here, we used DCF-DA as an independent measurement of intracellular RONS^30^. Figure 5A shows the brightfield microscopy image of RAW 264.7 cell line. Figure 5B–E represent fluorescence response of RAW cells, RAW cells treated with DCF-DA, RAW cells treated with LPS, RAW cells treated with both LPS and DCF-DA, respectively. The green signals from Fig. 5E confirm that RONS will be generated by treatment of LPS with RAW 264.7 cells. We used different concentrations of NAC as a free radical scavenger with high membrane permeability^31^. Figure 5F–H show decrease of green signal by increasing the concentration of NAC (0.1 mM, 1 mM, and 10 mM). Degradation of fluorescence signal is observed suggesting that the RONS are scavenged by the NAC. Figure 5I represents the quantification analysis of fluorescence data in Fig. 5B–H. RAW 264.7 cells treated with LPS and DCF-DA (RONS indicator) have increased fluorescence signal suggesting the generation of endogenous RONS. Figure 5I shows that adding NAC at 10 mM scavenged the RONS and decreased fluorescence.

Next, we measured RONS in RAW 264.7 cells with the photoacoustic probe. Here, cells were incubated with LPS as well as the probe (CyBA) with and without NAC (ROS scavenger). Figure 5J shows the MIP photoacoustic image for all six samples. RAW cells (control), RAW cells with the probe (+CyBA), RAW cells with LPS (+LPS), RAW cells with LPS, and CyBA (+CyBA + LPS), and RAW cells incubated with LPS/CyBA and various concentrations of NAC (0.1 and 10 mM) were placed in an agar phantom for photoacoustic imaging (Fig. 5J). There is low signal for RAW cells and RAW cells incubated with LPS but cells incubated with LPS have signal that is ~34-fold higher than the signal from RAW cells only incubated with CyBA; the NAC scavenger with concentrations of 0.1 mM and 10 mM decreased this signal 1.5- and 3.5-fold, respectively. This suggests that NAC scavenges the RONS leading to less photoacoustic signal (Fig. 5K). These results have been confirmed by fluorescence imaging (Fig. 5I).

### *In vivo* photoacoustic imaging evaluation of CyBA

**Figure 6:**
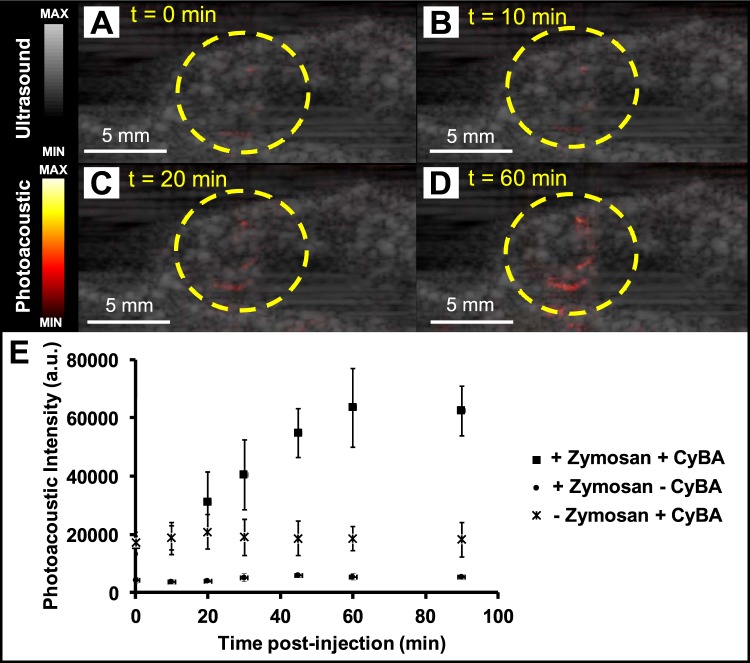
*In vivo* photoacoustic evaluation of CyBA. (**A**) Ultrasound/photoacoustic image at baseline. Ultrasound/photoacoustic image (**B**) 10, (**C**) 20, and (**D**) 60 minutes after CyBA injection. (**E**) Quantitative analysis of photoacoustic intensity as a function of time post-injection of CyBA. ~3.2-fold increase of photoacoustic intensity after 90 minutes of CyBA injection was observed. Photoacoustic intensity for only Zymosan (+Zymosan −CyBA) and only CyBA (−Zymosan +CyBA) are unchanged. The error bars represent the standard deviation of measurements among 3 mice.

We evaluated the ability of CyBA to measure inflammation in mice. Figure 6A–D demonstrate the ultrasound/photoacoustic image from injection location of probe and Zymosan at 0, 10, 20, and 60 minutes, respectively. These figures show the increase of photoacoustic signal due to tissue diffusion of the probe (CyBA) at edema area (lined yellow circles). Figure 6E represents the quantitative analysis of *in vivo* photoacoustic imaging evaluation of CyBA. These results revealed that photoacoustic intensity increased gradually and ~3.2-fold increase was observed after 90 minutes of CyBA injection. Figure 6E also shows unchanged photoacoustic intensity of only Zymosan and only dye.

## Discussion

Detection of H_2_O_2_ and ONOO^−^ in biofluids is important to medicine. The concentrations of H_2_O_2_ in healthy human urine are between 5 to 100 µM and depend on the age and gender of the subject^32^. H_2_O_2_ concentrations in plasma are as high as 35 µM^33^. Various levels of H_2_O_2_ (in some cases 100 µM or more) have been measured in the aqueous and vitreous humor^34^. The steady-state biologically concentration of ONOO^−^ is in the nanomolar to low micromolar level with a basal production rate of 0.1–1 µM min^−1^ ^35^. In an inflammatory microenvironment, the production rate will increase as high as 50–100 µM min^−1^ ^35,36^. We report here a sensitivity of 12.5 and 31 μM for ONOO^−^ and H_2_O_2_, respectively, using a LED-based photoacoustic imaging system (Fig. 3C,D).

LED-based photoacoustic imaging system can monitor RONS *in vitro* and *in vivo* using CyBA. LED-based photoacoustic imaging solves many clinical transition issues for photoacoustic imaging; thus, this technique and probe can be used for clinical monitoring of RONS for keloid diagnosis or in drug toxicity^37^.

Other RONS-sensitive photoacoustic probes with a wide range of sensitivities have been reported. Zhang *et al*. reported a bulky borane-doped nanoprobe with sensitivity of 0.1 µM to detect ONOO^−15^. Xie *et al*. used a self-assembly approach to measure 150 µM H_2_O_2_^14^. Kim *et al*. showed that their gold/silver hybrid nanoparticle could release Ag^+^ in presence of RONS such as ONOO^−^ and H_2_O_2_ with a sensitivity of 5 and 0.25 mM, respectively^38^. These results show that CyBA probe can measure H_2_O_2_ under both normal and inflammatory conditions. Although the detection of ONOO^−^ in steady state is not feasible using our designed probe and LED based system, we could measure ONOO^−^ under inflammatory conditions *in vivo*.

It is important to mention that most of reported RONS sensors are nanoparticles. One of the advantage of using small molecules sensor is that they can pass the membrane and access to all the cell’s compartments with a simple diffusion technique whereas nanoparticle usually will be trapped in the endosomes^39^. Therefore, this small molecule probe is more appropriate for *in vitro* imaging to monitor endogenous RONS in cells (Fig. 5).

The change in absorption spectrum or photoacoustic signal of the probe is a function of oxidation of boronate group to phenolic functionality^40^. The oxidation of the boronate group is quantitative and fast. Hence, imaging short-lived species such as RONS with chemodosimeters containing boronate-based probes is feasible in biological environments^41,42^. The hydroxyl functional group that results from oxidation governs the spectroscopic properties of these boronate-functionalized cyanine dyes^43,44^. The same chemical transformation is possible through direct oxidation in the presence of peroxynitrite^45^. The oxidation of boronate esters by peroxynitrite is a million times faster than hydrogen peroxide^46^. Thus, it is possible to detect peroxynitrite in real time inside the cells and to image peroxynitrite in animals^47^. This oxidation is then manifested via a bathochromic shift in absorption and increased photoacoustic signal to measure reactive oxygen species (Figs 2 and 3).

This probe was validated in buffer, plasma, and whole blood (Fig. 4), and ONOO^−^ in blood and plasma have been linked to cardiovascular diseases^48^. In the vascular compartment, the formation of ONOO^−^ is based on the reaction of nitric oxide (˙NO) with superoxide radical (O_2_^−^)^48^. The ONOO^−^ oxidizes plasmatic components in the intravascular spaces^49^. In addition, up to 40% of intravascularly-generated ONOO^−^ will diffuse and react into the red blood cells (RBC) before plasma^49,50^. Therefore, overproduction of either ˙NO or O_2_^−^ can cause intravascular formation of ONOO^−^.

## Conclusions

We measured RONS species in clinical samples with LED-based photoacoustic imaging. The LED-based photoacoustic imaging offers a significant reduction in the size and cost of optical components. While one limitation of LEDs is their 1000-fold lower power (leading to a need for higher repetition rates), this lower power is actually an advantage here. The higher power from Nd:YAG lasers photobleaches the dye, but the LED does not. Indeed, this work is the first report of LED-based photoacoustic molecular imaging. Future work will expand this work with LED-based photoacoustic to include higher frequency transducers and a larger portfolio of molecular imaging contrast agents.

## Supplementary information

Supplementary

## Data Availability

The datasets generated during and/or analyzed during the current study are available from the corresponding author on reasonable request.
