# Supplementary material for: Molecular imaging of oxidative stress using an LED-based photoacoustic imaging system
Source: Sci Rep. 2019 Aug 6;9:11378. doi: 10.1038/s41598-019-47599-2 (PMC6684596; doi:10.1038/s41598-019-47599-2)
Supplement: Supplementary file 1 — Supplementary [file 41598_2019_47599_MOESM1_ESM.docx]

**Molecular imaging of oxidative stress using an LED-based photoacoustic imaging system**

Ali Hariri^1^, Eric Zhao^1^, Ananthakrishna Soundaram Jeevarathinam^1^, Jeanne Lemaster^1^, Jianjian Zhang^4^, and Jesse V. Jokerst^1,2, 3^ *

1 Department of NanoEngineering

2 Materials Science and Engineering Program

3 Department of Radiology

University of California, San Diego, 9500 Gilman Drive, La Jolla, CA 92093, USA

4 Key Laboratory of Synthetic and Natural Functional Molecule Chemistry of the Ministry of Education, Modern Separation Science Key Laboratory of Shaanxi Province, College of Chemistry & Materials Science, Northwest University, Xi’an, China

* Correspondence and requests for materials should be addressed to [jjokerst@ucsd.edu](mailto:jjokerst@ucsd.edu).

**Figure S1.** Mass spectrometry data from **A)** CyBA, inset demonstrates the molecular structure with calculated molecular weight. **B)** CyBA after LED illumination, **C)** CyOH, inset demonstrates the molecular structure with calculated molecular weight. **D)** CyBA in presence of RONS (peroxynitrite (200 µM)).
